# Functional and Structural Variation among Sticholysins, Pore-Forming Proteins from the Sea Anemone *Stichodactyla helianthus*

**DOI:** 10.3390/ijms21238915

**Published:** 2020-11-24

**Authors:** Esperanza Rivera-de-Torre, Juan Palacios-Ortega, J. Peter Slotte, José G. Gavilanes, Álvaro Martínez-del-Pozo, Sara García-Linares

**Affiliations:** 1 Departamento de Bioquímica y Biología Molecular, Universidad Complutense, 28040 Madrid, Spain; erdto@dtu.dk (E.R.-d.-T.); juan.palaciosb1a@gmail.com (J.P.-O.); jpslotte@abo.fi (J.P.S.); jggavila@ucm.es (J.G.G.); alvaromp@ucm.es (Á.M.-d.-P.); 2 Department of Biochemistry, Faculty of Science and Engineering, Åbo Akademi University, 20500 Turku, Finland; 3 Department of Biotechnology and Biomedicine, Technical University of Denmark, 2800 Kongens Lyngby, Denmark

**Keywords:** actinoporins, cholesterol, cnidaria, leakage, sphingomyelin, venom

## Abstract

Venoms constitute complex mixtures of many different molecules arising from evolution in processes driven by continuous prey–predator interactions. One of the most common compounds in these venomous cocktails are pore-forming proteins, a family of toxins whose activity relies on the disruption of the plasmatic membranes by forming pores. The venom of sea anemones, belonging to the oldest lineage of venomous animals, contains a large amount of a characteristic group of pore-forming proteins known as actinoporins. They bind specifically to sphingomyelin-containing membranes and suffer a conformational metamorphosis that drives them to make pores. This event usually leads cells to death by osmotic shock. Sticholysins are the actinoporins produced by *Stichodactyla helianthus*. Three different isotoxins are known: Sticholysins I, II, and III. They share very similar amino acid sequence and three-dimensional structure but display different behavior in terms of lytic activity and ability to interact with cholesterol, an important lipid component of vertebrate membranes. In addition, sticholysins can act in synergy when exerting their toxin action. The subtle, but important, molecular nuances that explain their different behavior are described and discussed throughout the text. Improving our knowledge about sticholysins behavior is important for eventually developing them into biotechnological tools.

## 1. Venoms through History

Venoms have played an important role in human history, being not only subject of study but of fear and fascination. One of the very first examples to sustain this assertion, and probably one of the most widely known too, could be the biblical story starring Eve and Adam within the Book of Genesis (Genesis 3:1). A story with two principal venomous-related characters that triggered the expulsion from the Garden of Eden: a wise venomous snake and a poisonous apple tree (poisoned by *wisdom*). This allegoric story about obedience and temptation also reflects the antiquity of fear of the consequences caused by venom and poison manipulation. Snakes, in fact, are common venomous animals that provoke an innate animadversion in humankind. Somehow, this distress produced by these reptiles has been incorporated into our genomes through evolution to favor our survival. Anyway, this is only one of the many examples of the relationship of humans with venoms that we can find through history. The Greek philosopher Socrates was condemned to death by drinking hemlock, according to Plato’s *Phaedo* [1]. The Egyptian governor Cleopatra VII committed suicide by poisoning herself [2,3]. The roman emperor Nero had a poison expert as part of his court, Locusta, who might have been involved in the murder of Claudius and Britannicus [4,5]. Last, but not least, the Italian Borgia even developed the so-called art of poisoning [6]. All of these, and many other events described in ancient history, have increased our inherent apprehension and curiosity towards venoms and poisons.

Most venomous animals are small, compared to their predators. Venom is indeed an evolution tool that balances the prey–predator relationship further from physical features such as size, speed, or strength, putting the spotlight on the power of Chemistry. It is possible to find venomous animals in a wide variety of vaguely related phylogenetic origins: from vertebrates, like reptiles, amphibians, and even mammals, to invertebrates, like insects, arachnids, and some of the oldest animals’ phyla, like precisely cnidaria. All these animals use venom for predatory, defensive, or competitive purposes. Fear and fascination are not the only feelings awakened by all these organisms, as ancient civilizations also took advantage of venom effects to use them as drugs to treat different diseases. At the time, these treatments were based solely on experience, i.e., trial and error approaches. It was known that toxins could modulate diseases effects, even if their molecular basis was completely unknown. Now, such use can be supported by careful and detailed knowledge of their composition and mechanisms of action, opening a wide new field based on the use of toxins as a source of inspiration to build new biotherapeutics [7,8].

## 2. Venom Evolution

It is common to confuse the terms ‘poisonous’ and ‘venomous’, which are sometimes wrongly used indistinctly. Poisonous refers to agents that are passively delivered by contact, either through the skin or by ingestion, like some alkaloids produced by plants such as *Atropa belladonna*. On the other hand, venomous refers to those agents that require an active delivery from predator to prey, usually through specialized stinging structures like fangs (snakes), spines (fishes), nematocysts (sea anemones), or chelicerae (spiders), which cross the unspecific epithelium barrier. The prominent role played by venoms, and their corresponding defenses, in the predator–prey relationships makes them subject to a great evolutionary pressure. Continuously, and unwillingly, each part selects the most effective variants of its counterpart in a never-ending arms race [9,10,11]. Resulting venom mixtures are then complex, and usually adapted to attack a wide range of prey through different approximations, like a variety of molecular targets at the plasma membrane.

A generally accepted hypothesis about how venom components can originate assumes that a proto-toxin gene could duplicate. Then, if mutations take place, a new isoform would appear. If the functionality of this isoform (or isoforms, if multiple duplication and later mutation events take place) presents some advantage when expressed in an exocrine manner, natural selection will likely drive the specialization of the gland and its protein(s)/toxin(s) into a toxin-delivery system. Due to this duplication–neofunctionalization mechanism, it is usual to find venom toxins genetically structured as multigene families [11,12,13,14,15,16,17,18,19]. This theory seems feasible because most proteins present in venoms execute nontoxic physiological functions when produced in other tissues. For example, phospholipases A2 (PLA2s), which are enzymes that catalyze the hydrolysis of glycerophospholipids at the *sn*-2 position, releasing free fatty acids and lysophospholipids, are currently involved in cell signaling. Simultaneously, PLA2s are also frequent venom toxins that damage the plasma membrane of their cellular targets. The results of their enzymatic activity, some of which are detergent-like molecules, affect membrane curvature, triggering stress and inflammatory signals [20]. Venoms would then also be a good example of convergent evolution, since similar venomous systems with independent origins can be observed in far related species. This is the case of many other biological systems, such as those responsible for echolocation in bats and cetaceans, wings in insects and birds, or, maybe the most well-known example, eyes, which can be found in cephalopods, vertebrates, and even cnidaria [21].

Venoms are always complex biochemical cocktails containing salts, peptides, proteins, and small metabolites acting as bioactive compounds. Upon delivery, the toxins interact with cellular structures causing damage, altering physiological and signaling processes. This mechanism implies an interaction between the toxic components of the venom and specific cellular structures of the target. Delivery systems, like chelicerae in spiders and nematocysts in cnidarians, run through the first barrier encountered in the prey, the epithelium. These piercing structures deliver toxins closer to their final molecular target. The process is facilitated by specialized enzymes (like metalloproteases, for example) that digest components of the extracellular matrix and other scaffold structures present in the cell interstitial space. Another common target of small nonproteinaceous metabolites, like histamine or serotonin, is the coagulation cascade. They interfere by altering coagulation time or provoking vasodilatation, thus triggering fatal consequences for the attacked organism.

The most obvious second line of cell-defense is the boundary defined by the plasma membrane. This boundary, composed of many different lipid and proteins, defines the cell as a compartment separated from the extracellular environment, establishing a well-defined frontier between the intra and extracellular contents, which display different physicochemical properties and composition [22]. Thus, the most widespread structures encountered by venomous cocktails on any type of prey are the transmembrane and peripheral proteins integrated within the lipid bilayer, as well as the lipid membrane itself. Toxins that disrupt their function commonly target ion channels. Consequences of this molecular action derive from the ion imbalance developed between the inside and the outside of the cell, usually producing intense pain, paralysis, massive release of neurotransmitters, and even cell death by membrane depolarization. Another type of membrane-targeting toxin frequently appearing in venoms does not affect its protein constituents but directly the integrity of the whole structure, maintained mostly by phospholipids. This is the case of the aforementioned phospholipases or the widespread pore-forming proteins (PFPs) [12,23,24,25,26,27,28,29,30,31,32]. Given the critical importance of the plasma membrane for cell viability, its disruption usually has fatal consequences that, in many cases, imply cell death by osmotic shock. Finally, some other venom toxins target intracellular structures, disabling key processes like protein production or oxidative phosphorylation in mitochondria [33], for example.

As stated before, ancient civilizations used venoms as treatments for a wide variety of diseases. Within the context of the pathologically affected metabolic pathways, we now know that some toxins have the ability to establish high affinity interactions with specific molecules, balancing or modulating disease effects. Nowadays, it is possible to study venom components in detail and reveal the molecular features of these interactions, allowing to synthetize and test native or modified molecules that can serve as therapeutic treatments [7].

## 3. Pore-Forming Proteins

PFPs are a group of toxins whose activity precisely relies on the disruption of the lipid membranes by forming pores. These kinds of toxins escape to the archetypal biochemical classification that sorts proteins into water-soluble or membrane macromolecules [12,23,24,25,26,27,28,29,30,31,32,34,35,36,37]. They are produced as soluble monomeric proteins and remain stably folded and water-soluble but, upon interaction with a membrane receptor in the target cell, undergo a molecular metamorphosis to become an oligomeric transmembrane assemble, that forms a pore within the membrane core. Such receptor can be a membrane bound or transmembrane protein, but also a sugar, or a specific lipid [35], as is the case of sea anemone actinoporins [38,39,40]. The specific pore-formation mechanism driving this transformation largely depends on the toxin, but all of them take advantage of their increased local concentration, which is consequence of restricted diffusion on a bidimensional plane once the protein is bound to the membrane, facilitating oligomerization [41]. Depending on the size and physicochemical properties of the resulting channel, the final pore can be permeable to different ions, or even to small proteins or peptides [27,42,43]. As stated above, in most cases, the outcome is cell death by osmotic shock.

There are many ways to categorize PFPs. The most extended classification is based on the nature of the secondary structure of the protein stretch or domain that builds the pore walls. Hence if they are formed by α-helices, they are classified as α-PFPs; and, if they are defined by β-strands, the proteins are considered β-PFPs [24].

PFPs are implicated in both physiological and pathological functions. They are produced by many pathogenic bacteria, playing a major role as virulence factors [35,44,45,46,47,48,49], but they can also be found in more complex organisms such as mammals, where they usually develop physiological functions. Most probably, the best examples in mammals would be the complement system (MACPF) [44,50,51,52,53], involved in the action of both the innate and adaptive immune systems of vertebrates, and the BAX/BAK [54,55,56,57,58,59] protein families, responsible for apoptosis regulation at the mitochondrial level.

The large and widespread PFPs group is made of highly damaging molecules. As stated before, they attack a primordial feature of any living cell: the plasma membrane. PFPs present different mechanisms to target their objective. The most common ones are the interaction of a specific lipid, like cholesterol (Chol) [53,60,61,62,63,64,65,66,67,68,69,70,71,72,73] or sphingomyelin (SM) [12,25,26,44,69,74,75,76,77,78,79,80,81,82,83,84,85,86,87,88,89,90,91], or recognition of a specific membrane protein receptor [32,92,93,94,95,96]. It is common to find collections of highly prey-specific toxins as part of multigene families resulting in an extended range of different targets [97]. PFPs targeting some highly abundant lipids, such as the mentioned SM or Chol, may not seem highly specific. However, as part of the venom mixture, they constitute key elements to pursue a wide range of different enemies or prey, also including the multigene-family feature. Furthermore, Chol and SM are indeed specific constituents of most vertebrates’ cellular membranes. It is also remarkable how PFPs toxic action usually displays fast kinetics behavior [86,87,91,98], explaining why they can be used for both predatory and defensive purposes [9,11,99,100,101].

## 4. Cnidarian’s Pore Forming Proteins

**Figure 1:**
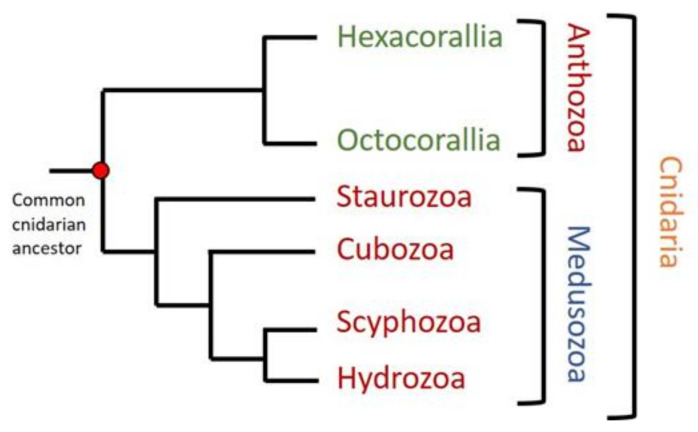
Phylogenetic tree of cnidaria phylum. Two different groups can be distinguished in cnidaria: Anthozoa, containing Hexacorallia and Octocorallia subclasses, and Medusozoa, including Staurozoa, Cubozoa, Scyphozoa, and Hydrozoa. Figure modified from [103,105].

Cnidaria is the oldest linage of venomous animals. Its study from the evolutionary and phylogenetic point of view is interesting in order to fill the gaps in most general genetic and phylogenetic studies focused not only on venom evolution [11], but also in the appearance of the nervous system or the generation of bilateralism [102,103]. This phylum comprises about 10,000 different aquatic species, most of them living in salt water. The phylum contains two main groups. Anthozoa is a class that includes sea anemones and corals, species living as sessile polyps. It is subdivided into the subclasses Hexacorallia and Octocorallia, according to their different radial body symmetries. The other group is the subphylum Medusozoa, which comprises four other classes: Hydrozoa (*Hydra* and colonial polyps), Scyphozoa (true jellyfishes), Cubozoa (box jellyfishes), and Staurozoa (Stalked jellyfishes) (Figure 1). Medusozoa have a life cycle including polyp and medusa stages, while Anthozoa occur only as polyps [103,104,105]. Most of these animals are venomous and clinically relevant. The consequences of their sting vary from nonhazardous symptoms like burning feeling, itching, and redness, typically caused by sea anemones and corals [81], to important grievances like severe pain, cardiovascular distress, and loss of consciousness caused by the most dangerous species like the Australian box jellyfish (*Chironex fleckeri*) [106,107,108].

Venomous animals have stinging venom administration systems. These specialized structures are responsible for traversing the epidermal barrier and injecting the venom in the prey. In sea anemones, nematocysts perform this function. Nematocysts discharge their venomous load upon pressure activation of specialized cnidocyte cells [9,10]. Animals of the cnidaria lineage lack a centralized venomous system. In tentacles, cnidocytes are present surrounding the oral disk too, in order to paralyze the prey. They also appear within the column base, as part of specialized structures used for inter- and intraspecific competition [109,110].

Like most animal venoms, cnidarian toxic load also contains salts, small metabolites, peptides, and proteins. Within the last group, quantitatively speaking, PFPs constitute one of the most important toxins in cnidarians [19,111,112,113], in both quantitative and toxic terms. The already mentioned Cubozoan Australian Box Jellyfish (*Chironex fleckeri*), for example, owes its extreme venomous potency to two highly abundant PFPs, CfTX-1 and 2, which display potent hemolytic activity [107,114,115]. In fact, sea anemones host some of the most important and well-studied families of PFPs: Actinoporins, the main subject of this review.

## 5. Sea Anemone Actinoporins

Actinoporins are a well-studied group of α-PFPs. Sea anemones produce them as part of their venom, which contains several other classes of cytolysins. Regarding their structural and physicochemical properties, actinoporins are relatively small proteins (around 20 kDa) that usually exhibit a basic isoelectric point (around 9) and lack cysteine residues [25,26,36,110,116]. They are synthesized as immature products with a pre-propeptide region [17,19]. Maturation takes place by cleavage of the pre-propeptide upon secretion to the cnidocyte lumen [17]. As many other venom protein components, they also appear as multigene families. A single sea anemone individual has several genes for similar but not identical actinoporins [12,14,17,18,19,26,117,118]. As explained above, this multiplicity is probably generated by gene duplication, as an advantage to fit into new ecological niches. The driving force would be the predatory and defensive competition for survival [17,119]. Actinoporins do not need a protein receptor to bind to the membrane. Instead, a specific lipid is used, SM [38,79,81,82,87,91,116,120]. In addition to SM, Chol is not strictly needed but its presence seems to strongly favor pore-formation [86,87,88,91,121,122], a very important feature given the high percentage of this sterol in vertebrate membranes. Indeed, actinoporins represent a simple, and therefore optimum, model to study the biophysical aspects of the transformation of a water-soluble protein into a membrane-integrated structure.

**Figure 2:**
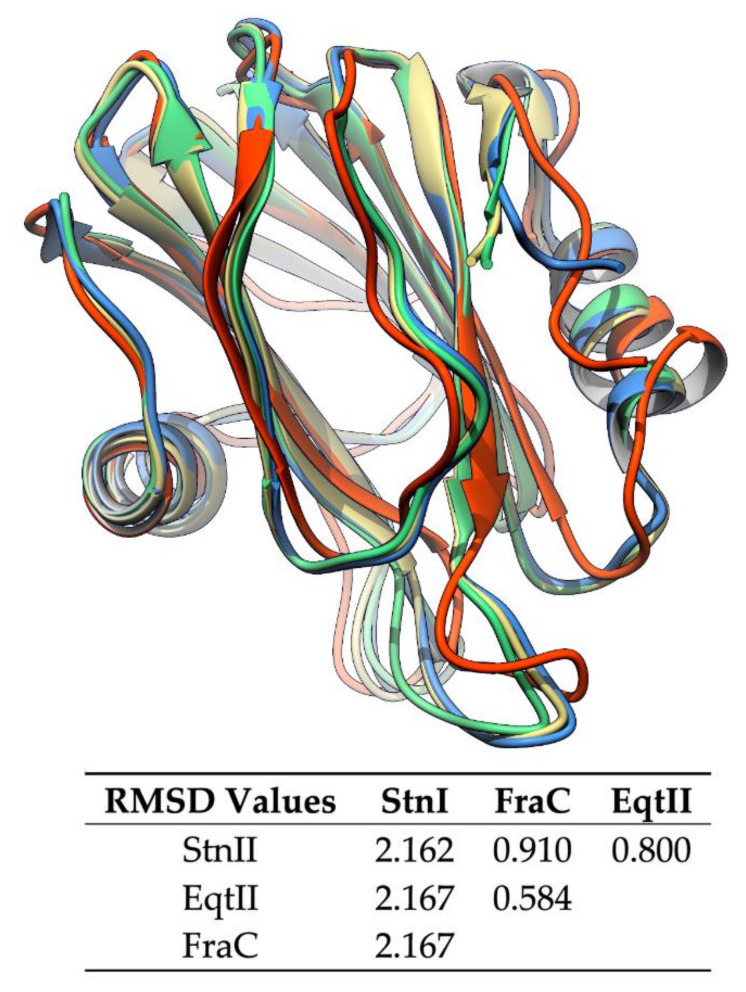
Overlapping structures of the solved actinoporin three-dimensional structures. Sticholysins I (StnI) (2KS4, red) and II (StnII) (1GWY, blue) from *Stichodactyla helianthus*, fragaceatoxin C (FraC) (3W9P, green) from *Actinia fragacea*, and equinatoxin II (EqtII) (1IAZ, yellow) from *Actinia equina*. They share a common characteristic fold, a β-sandwich core flanked by two α-helices. The RMSD values are shown in the table below. Values are much higher when comparison is made against StnI because it is a structure obtained by NMR [127,129], while the other three are crystalline structures [123,125,128].

Actinoporins have been found in at least 20 venomous sea anemone species [119]. They are intra and interspecifically conserved, presenting sequence identity values above 90% in some instances [32,36,87]. This is reflected by a conserved characteristic three-dimensional protein fold [123,124,125,126,127,128], which consists of a β-sandwich core flanked by two α-helices (Figure 2). Despite their similarity in terms of sequence and global structure, they display different toxic properties, showing variability regarding membrane binding affinity and pore formation. This variability is assumed to be an advantage in terms of expanding the range of prey that a sea anemone can attack, in addition to the possibility of establishing synergic interactions [118].

**Figure 3:**
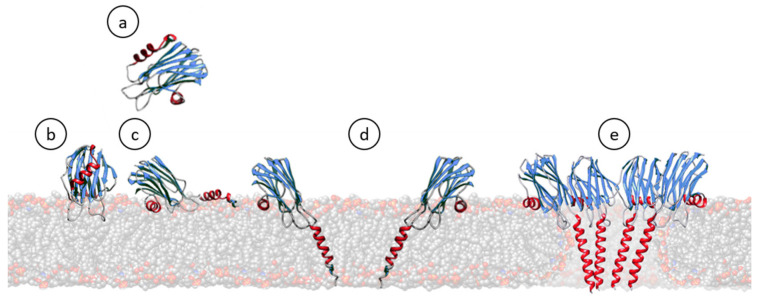
Cartoon schematic representation showing most of the steps generally accepted for the pore formation mechanism of actinoporins. In solution, they remain soluble and stably folded (**a**) [26,110,124,127,136,137,138,139]. Upon interaction with a lipid membrane containing SM (**b**), their N-terminal α-helix stretch is detached and extended, shortly laying parallel to the membrane (**c**) [25,26,82,85,87,91,116,140,141,142,143]. Then, monomers oligomerize and insert this N-terminal α-helix, now about 30 residues long, within the hydrophobic membrane core (**d**) [34,144,145,146,147,148,149]. The existence of such an intermediate is one of the most controversial issues, depending on the degree of acceptance of the evidence about the real existence, or not, of pre-pore assemblies [132,133,150]. Finally, a cation selective channel is established (**e**) [39,116,128,136,144,151].

All well-characterized actinoporins seem to follow the same mechanism of pore formation. Upon interaction with lipid membranes containing SM, the protein with the corresponding water-soluble conformation binds to the bilayer, extends its N-terminal stretch, detaching it from the β-sandwich core, to yield a much longer and now amphipathic α-helix. This helical segment then lies parallel to the membrane surface while protomers begin to establish protein–protein interactions in order to oligomerize. Simultaneously, the extended N-terminal α-helix penetrates the membrane hydrophobic core, finally forming a cation-selective pore [25,26,31,36,82,122,130] (Figure 3). This mechanism is subject of quite consensus. However, the specific order of the steps leading to the final pore formation [31,34,128,131,132], the implication of ‘pre-pore’ structures during the process [31,132,133], and the stoichiometry and detailed pore structure [31,125,126,128,131,134,135], are still controversial.

**Figure 4:**
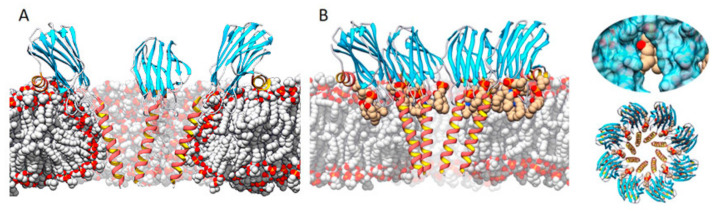
Two of the main models representing the pore structure of actinoporins. (**A**) A tetrameric one in which the membrane adopts a toroidal shape around the pore walls (made using StnII model 1GWY fitted to the structure of FraC from model 4TSY), and (**B**) an octameric lipid–protein structure (FraC pore, PDB ID: 4TSY) in which lipids (carbon atoms in tan color) are accommodated through pore wall fenestrations. Inserts on the right show a close-up of a lipid on a fenestration (top) and a top view of the octameric complex (bottom). Adapted from [32]. The α-helices are depicted in red and gold, β-sheets in blue, nonperiodic structures in grey. Bulk lipids are depicted in grey (carbon atoms) and red (oxygen atoms).

Three transmembrane pore structures of actinoporins have been solved so far, though two of them are of very low resolution [125,126,134]. Attending to the chronological order of publication, the first one suggested a tetrameric toroidal protein–lipid structure [133,134,135,152], where phospholipid heads would play a key role in lining the lumen of the pore channel (Figure 4A). Nonetheless, some years later, a highly detailed model of an actinoporin’s pore was solved at atomic resolution from a lipid-containing crystal [128] (Figure 4B). In this model, lipids appear to play an important role in configuring the channel walls, being accommodated through fenestrations between the helices (Figure 4B). Considering the structures solved so far, and taking into account the kinetic studies performed by some other authors [31,36,87,131,139], it can be speculated that the structures described so far might represent different possible, condition-dependent, thermodynamic equilibriums or, in much simpler words, different snapshots of the same movie.

**Figure 5:**
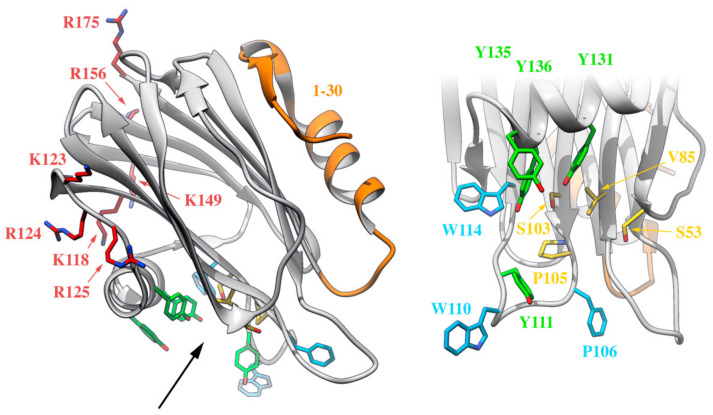
Four different functionally relevant regions can be distinguished in the water-soluble monomeric actinoporins, as depicted on this representation of the three-dimensional structure of StnII (PDB: 1GWY). The N-terminal stretch (in orange), an array of exposed and basic amino acids (in red and blue), a cluster of aromatic residues (in light blue, except the Tyr residues also belonging to the POC site, which appear in green), and the POC binding site (in yellow or green). Residues taking part in both the cluster of aromatic residues and the POC binding site are highlighted in green. The black arrow on the left panel indicates the point of view of the right panel, which is a close-up of the cluster of aromatic residues and the POC binding site.

It is possible to differentiate at least four well-defined functionally relevant regions in actinoporins: a cluster of aromatic amino acid residues, an array of basic residues, at least one phosphorylcholine (POC) binding site, and the N-terminal, α-helix containing, stretch comprising approximately the first 30 residues (Figure 5). The exposed cluster of aromatic residues has an important role in the very first membrane binding steps. Mutations in this region result in less hemolytic variants with reduced membrane affinity [141,153]. The array of basic amino acids has also been proposed to be critical in the initial steps of membrane recognition via interaction with negatively charged regions of the lipid head groups [26,124]. Within this idea, negatively charged phospholipids would also contribute to favor nonlamellar phases, modify bilayer curvature, and increase cation selectivity [75]. The POC binding site seems responsible for SM head recognition. It is partly hydrophobic and partly hydrophilic with the interesting feature that some of the participating residues are also members of the aromatic cluster. During the interaction, the positively charged POC seems to be stabilized by cation-π interactions with the aromatic ring of two tyrosine residues, whereas the phosphate group interacts with the phenolic hydroxyl groups of these residues and is probably stabilized by the cationic side chain of several basic amino acids [26,125]. It also establishes key hydrogen bonds with SM [85,86,87,88,91]. The latest octameric pore structure of FraC proposes the existence of additional lipid-binding sites, presumably for SM. Though considered as highly probable, their existence has not been proved with enough detail yet [128]. Finally, the N-terminal 30 residue-stretch, comprising one of the two α-helices, is the region that detaches from the β-sheet core to form the pore walls (Figure 2, Figure 3 and Figure 4). It is a crucial segment for pore formation, but it does not appear to be involved in membrane attachment [146,154]. This region is precisely the most variable region within actinoporin sequences. Slight differences in this stretch seem to have consequences regarding conductivity properties of the pore, maybe accounting for differences in toxicity [155,156] (Figure 5).

## 6. Sticholysins I, II, and III

*Stichodactyla helianthus,* commonly known as the sun anemone, is a carpet-like venomous sea anemone from the Caribbean Sea. It produces at least three actinoporin isoforms: StnI, II, and III. The two former ones, StnI and II, are quite abundant and easily detected in the crude venom and can be purified to homogeneity in large amount from sea anemone homogenates [81,157,158,159,160,161]. The third one, StnIII, which was discovered only after the *de novo* transcriptomic analysis of *S. helianthus* [19], is apparently expressed in much less quantity, and only recently has it been characterized [162].

**Figure 6:**
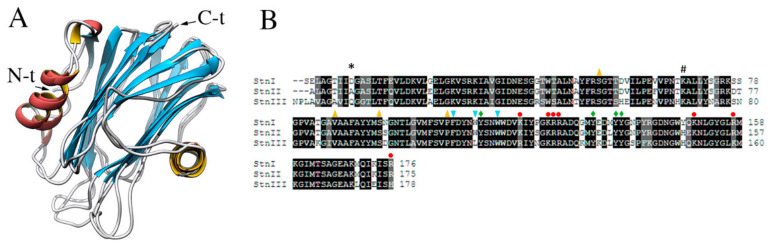
(**A**) Three-dimensional structures of StnI (2KS4) and II (1GWY). (**B**) Sequence alignment of StnI, II, and III. Identical residues are highlighted in black, those with similar/conserved chemical properties appear grey, and nonconserved residues remain backgrounded in white. Black asterisk (*) indicates StnI Asp9 (Ala8 and Gln11 in StnII and StnIII, respectively). Black hashtag (#) indicates a conserved Lys residue in StnI, StnII, and StnIII, occupying positions 68, 67, and 70, respectively. As indicated in Figure 5, red circles highlight residues that conform the array of basic amino acids. Light blue arrowheads pointing down indicate the residues taking part of the cluster of aromatic residues, yellow arrowheads pointing up mark the amino acids that are part of the POC binding site, and green diamonds indicate residues belonging to both the cluster of aromatic residues and the POC binding site. These residues appear conserved in all three Stns except for those corresponding to StnI Trp111, Lys119, and Arg176 which, only in StnIII, are Leu113, Arg121, and His178. This figure has been modified from [162].

StnI and II are very similar proteins that share 93% of their amino acid sequence but still show quite different membrane binding affinities and, subsequently, pore forming and hemolytic activities [25,75,81,87,127,163,164]. StnII is about four-fold more effective than StnI against sheep erythrocytes in causing hemolysis. In agreement with their high identity, both show almost identical three-dimensional structures (RMSD 2.162, Figure 2) [125,127] (Figure 6A). On the other hand, at the protein sequence level, StnIII is only 76% identical to StnI and 77% to StnII (Figure 6B). Phylogenetic analysis revealed that StnIII is in fact more closely related to other sea anemones species’ actinoporins rather than to the other two Stn isotoxins produced by *S. helianthus* [19], an interesting feature from an evolutionary point of view. Sequence conservation is still similar enough to allow the prediction of StnIII’s three-dimensional structure [19,162], which results in a conformation perfectly compatible with the standard fold of actinoporins [125,127]. This prediction was further supported by the observation that the three Stns considered show practically indistinguishable far-UV circular dichroism spectra [162].

Despite StnIII maintaining the well-preserved fold of all actinoporins, it still shows important structural and functional differences. For example, it is significantly less thermostable and, while its membrane binding affinity for DOPC:SM:Chol (1:1:1) model vesicles was only marginally smaller than for StnI, it was almost seven-fold lower than that of StnII [136]. Considering that membranes are highly dynamic structures constituted by molecules which only establish rather weak, noncovalent, interactions among them, ITC measurements allow estimation of the average mean value of lipids (n) affected by the proteins binding to the bilayer [141,165]. This does not necessarily imply specific direct contact between all these n lipid molecules and the protein, but how far away the membrane lipids are perturbed by protein binding. Within this idea, another significant difference was the lower number of lipid molecules affected by StnIII binding when compared to the values obtained for the other two Stns [141,162]. Its functional characterization showed that the critical concentration needed to form active pores was higher than for either StnI or II, showing a distinct behavior when oligomerizing on membrane surfaces. Altogether, these observations [136] suggest differences in the mechanism of action of this recently discovered actinoporin. The necessity of a higher StnIII concentration for equal activity against erythrocytes or model lipid vesicles, and other previously mentioned differences in behavior, could be compatible with StnIII oligomerizing into a final membrane structure whose stoichiometry would be different from that corresponding to StnI and II pores. Nevertheless, this is a prediction that remains to be proven.

## 7. The N-Terminal Stretch of the Different Sticholysins

As with many other actinoporins, most differences among StnI, II, and III are concentrated in their N-terminal segments, comprising approximately their first 30 amino acid residues and including one of their characteristic α-helices (Figure 5 and Figure 6). These differences involve changes between hydrophobic and charged residues in many cases, shifting the net hydrophobicity of their sequences, a feature that might in part account for their differences in toxicity [19,162,166].

The N-terminal end of StnIII deserves attention because it is two or three residues longer than the corresponding region in StnI and II, respectively (Figure 6), and shows a Pro residue at its second sequence position (Figure 6). These observations are interesting within the context that it has been proved that in StnI and II this N-terminal stretch length has been optimized for spanning a membrane whose bilayer thickness would correspond to membranes whose main component is dioleyl-phosphatidylcholine (DOPC) [148]. The comparatively longer N-terminal stretch of StnIII suggests the possibility of a different membrane thickness preference, thicker in comparison with the optimum value determined for StnI and StnII. The hydrophobicity of this N-terminal helix is also lower for StnIII, suggesting a minor tendency to cross the membrane than StnI and II [19,148,162,166] maybe also contributing to explain its lower hemolytic activity. Interestingly, DOPC-like membranes are some of the most common in fishes [167], which would be the preferred prey of sea anemones in nature.

Residues corresponding to StnI Asp9 are also of remarkable importance regarding the role of the N-terminal α-helix in pore formation. This position is occupied by Ala8 in StnII and Gln11 in StnIII (Figure 6). As suggested above, these changes shift in the hydrophobic profile of the α-helical stretch responsible for wall pore lining. Work developed with peptides mimicking the first 30 amino acids of StnI and II supported the conclusion that the different toxic activity exerted by these isoforms was partly due to the different hydrophobicity of the α-helices [156]. However, when this hypothesis was tested with the full-size proteins, considering the possible specific interactions between the N-terminal stretches and the β-sandwich core, it was shown that an exposed salt bridge established between Asp9 and Lys68, located at the β-sandwich core, was key for modulating the pore-forming activity of this actinoporin [166]. According to these results, this salt bridge would impair α-helical detachment, disturbing the extension process over the membrane that finally leads to cross the hydrophobic core and build the pore. The differences observed for StnI Asp9 in StnII and III would render absent the key salt-bridge observed in StnI between Asp9 and Lys68 (Lys68 being the only residue of the pair that is conserved in all three proteins) (Figure 6) [162,166]. Thus, the presence of Ala instead of Asp in the equivalent position of StnII would avoid the establishment of that interaction, easing detachment of the N-terminal α-helix, which could then explain its much higher pore-forming efficiency than the StnI version [87,166]. The equivalent residue in StnIII is a Gln (Figure 6). Although not charged, it still displays enough negative charge density to be able to interact with the corresponding conserved Lys, maybe explaining the lower hemolytic activity of StnIII too [162]. This is, of course, still speculative but it seems reasonable to admit that it is also arguable that the hemolytic activity must depend on several other, still to be discovered, molecular interactions. In summary, the hydrophobicity profiles of the N-terminal segment of Stns are important for effectively crossing the membrane. However, another crucial structural feature, leading to a greater pore damaging efficiency, seems to be the easiness of this segment to detach from the β-sandwich core, which is easier in StnII than in StnI or III because of the lack of the mentioned electrostatic interaction impairing its movement.

## 8. The Nature of the Sticholysins’ Functional Pore

Release of aqueous contents from model lipid vesicles has been a standard procedure to evaluate pore formation efficiency by actinoporins for the last few decades [40,75,86,87,88,91,118,122,136,148,166,168,169,170,171,172,173,174]. However, regardless of the probe of choice, the results reported show that the action of Stns is not able to empty the vesicles completely. This is hard to explain if StnII pores were to be stable and always leaky for the probes used. To address this question, we used a variety of fluorescent probes, including rhodamine 6G or Tb^3+^, to test the permeability of StnII’s pores [98]. The results indicated that those probes were in fact too large, and that the standard method in the field would be reporting StnII-induced transient permeation of the membrane rather than the passage of solutes through the stably assembled pores. In order to evaluate the permeability of these structures, we also used a somewhere else described [175] 1,2-dioleoyl-sn-glycero-3-phosphoethanolamine-N-(7-nitro-2-1,3-benzoxadiazol-4-yl) (POPE-NBD)-dithionite assay [98], which showed that the final pores were in fact open. Thus, everything indicates that the stable actinoporins’ pores are open in spite of the classically plateaued release curves obtained.

When this work was completed, it was concluded that size, rather than charge, was the key factor for discriminating passage through actinoporins pore [98]. This assertion does not contradict the well-established fact that it is a cation specific channel [136,151,168,171,176], especially in kinetic terms, considering the asymmetry of cation flow through the channel. Leakage observed using model lipid vesicles and the archetypical fluorescent probes generally employed in the field would only reflect transient instability of the membrane, probably mediated by dynamic, not completely assembled, pore intermediates, and initiated by helix insertion in the membrane. These intermediates, however, are of great significance for the molecular mechanism leading to the final, thermodynamically stable assemblies of the still controversial structure of actinoporins’ pores. Besides the proper pore, these stages of pore formation would greatly increase membrane permeability, which would imply serious damage in the case of living cells. In our opinion, calcein can still be used to study leakage induced by actinoporins, but results should be interpreted with caution in light of new evidence shown [98]. R6G can be a better probe in terms of sensitivity but, as calcein, is still too big for actinoporin’s pores. Formation of competent final and thermodynamically stable pores should be checked using the mentioned (POPE-NBD)-dithionite based assay [98].

## 9. Synergy among the Different Sticholysins

As stated above, actinoporins appear as multigene families that give rise to different protein isoforms in the same individual, displaying high sequence identities but functional differences. The evolutionary advantage of producing such similar isotoxins is not fully understood. A few years ago, StnI and StnII were used to show that actinoporin isoforms can act in synergy [118]. Through hemolysis and calcein releasing assays, it was revealed that mixtures of StnI and StnII are more lytic than the same amounts in equivalent preparations of the corresponding isolated isoforms acting separately. In fact, trace amounts of StnII enhanced StnI binding affinity to cell membranes, driving a dramatic improvement of hemolytic activity [118]. Furthermore, StnI and StnII could be chemically cross-linked at the membrane showing the formation of stable heteropores [118].

**Figure 7:**
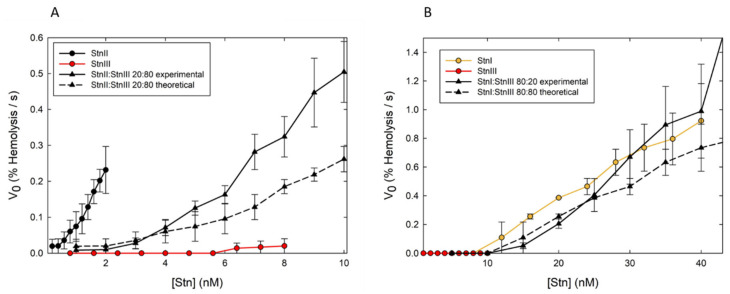
Synergic effect on hemolysis. Maximum hemolytic rate values (expressed as percentage of hemolysis per second) are represented as function of protein concentration (in log scale). (**A**) StnII (black dots–solid line), StnIII (red dots–solid line), a StnII:StnIII (20:80) mixture (black triangles–solid line). (**B**) Same as in (**A**), but now the proteins employed were StnI (orange dots–solid line), StnIII (red dots–solid line), and StnI:StnIII (80:20) mixtures (black triangles–solid line). In both panels, the black triangles–dashed lines were obtained as the arithmetic addition of the rates obtained with the individual proteins for the real concentration of each one in the different mixtures employed. Values are average of *n* = 3 ± SEM. Hemolysis assays were performed in 96-multiwell plates at 25 °C and exactly as described previously [118]. Briefly, erythrocytes from heparinized sheep blood were washed in 10 mM Tris buffer, pH 7.4, containing 145 mM NaCl, to a final A_655_ of 0.5 when mixing equal volumes of the cell suspension and buffer. The hemolysis was followed as a decrease in A_655_ after addition of the erythrocyte suspension to different final concentrations of protein. An Expert 96 microplate reader (Asys Hitech, GmbH, Eugendorf, Austria) was employed to measure the absorbance. The value obtained with 0.1% (w/v) Na_2_CO_3_ was considered as 100% hemolysis.

The discovery and subsequent characterization of StnIII allowed pursuing this study with this new sticholysin. Such synergy also takes place, at least, between StnII and III (Figure 7A). On the other hand, whether StnI and III show synergy could not be solved by looking only at the combination of their hemolytic activities. Both proteins show very similar hemolysis rates within the range of protein concentrations studied (Figure 7B). Since StnII and III show synergy, it can be speculated that, most probably, they also form mixed heteropores and that the synergistic effect between them would also occur at the membrane-binding step, with StnII facilitating StnIII binding. This observation reinforces the more general hypothesis that the main reason for the presence of several actinoporin isoforms in most of the studied sea anemone venoms would be to improve their versatility in defense and/or attack responses in their natural environment. These results confirmed that StnIII is another interesting piece in the puzzle of how *S. helianthus* modulates its venomous activity.

These StnI-StnII and StnII-StnIII synergic behaviors seem to be one of the explanations for the complexity and regulatory capacity of these toxins’ action; potentially increasing the range of species *S. helianthus* can capture or defend itself from. However, findings on eventual target specificities would be of great help to explain the proposed evolutionary advantage of this actinoporins’ multiplicity. This possibility would translate into more versatile defense and/or attack responses in their natural environment. Taken together, all the results have sound consequences in terms of the biological functionality of actinoporins and suggest that they could represent a more general strategy employed by other PFPs.

## 10. The Influence of Cholesterol

Chol is known to enhance the activity of StnII [121,122], although this activation is not Chol specific but rather sterol specific [86,88]. This is in good agreement with the heterogeneous sterol composition of marine invertebrates [177], some of which may constitute prey of *S. helianthus*. It has been also shown how membrane binding affinity for DOPC:SM:Chol (1:1:1) vesicles was similar (within the same order of magnitude, but still about five-fold higher for StnII) for all three sticholysins and, most importantly, ≈100-fold higher when compared to that of the vesicles without Chol [87,162]. In the Chol-containing system, however, StnII is much faster than StnI at producing calcein leakage, a result that also agrees with the differences observed in hemolytic activity [87,162]. Erythrocytes, after all, have a high content of Chol in their membranes (≈30–40%). Human red blood cell membranes are 1.5–2.0-times richer in cholesterol compared with any other cell in the body [178], for example. This ability was not interpreted as a higher affinity for the membrane but rather an improved ease of diffusion, oligomerization, and penetration of the bilayer, given that in the presence of Chol membrane binding would not be the limiting step [87].

**Figure 8:**
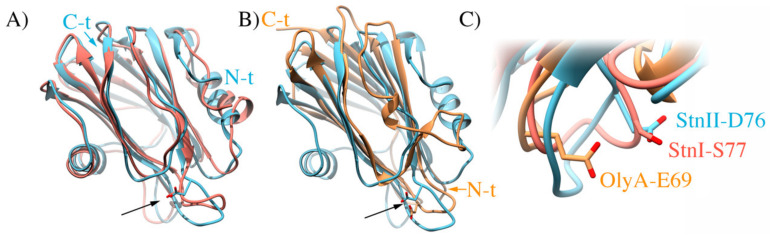
(**A**) Overlapping structures of StnI (salmon; PDB ID 2KS4) and StnII (cyan; PDB ID 1GWY). The arrow points at residues D76 of StnII and S77 of StnI. (**B**) Overlapping structures of StnII (cyan) and OlyA (orange; PDB ID 6MYI). The arrow points at residues D76 of StnII and E69 of OlyA. (**C**) Close up to the compared position, showing the sidechains of the mentioned residues as sticks. Colors as in (**A**,**B**).

It is intriguing, however, why StnII is especially sensitive to Chol. About two years ago, it was published how the toxin ostreolysin A (OlyA), a pore-forming protein from the edible oyster mushroom (*Pleurotus ostreatus*) that only binds to membranes when they contain both SM and Chol [179,180], based its Chol specific recognition on the strategic location of only one of its glutamic acid residues (Glu69) [90]. As a side effect of this work, it was also confirmed that SM can adopt two distinct conformations, depending on the presence or not of Chol in the membrane [120,181,182]. Although not an actinoporin, OlyA still shows high structural similarity to the structure of actinoporins (Figure 8). Inspection of the three-dimensional structures of OlyA and StnII revealed the presence of an Asp residue (Asp76) in actinoporins at an equivalent position of that of Glu69 in OlyA.

This residue is absent in StnI, where the corresponding amino acid is a serine (Figure 8), suggesting that this StnII Asp76 could be a molecular reason justifying the observed differences between both sticholysins regarding their different behavior in the presence of Chol [87,162]. In order to test this hypothesis, studying the membrane interaction properties of a (yet to be produced) StnII-D76S mutant [183] in both the presence and absence of Chol might be a good approach to solve this different sterol recognizing behavior of sticholysins.

In order to further explore how bilayer lipids affect, or are affected, by StnII, a multiprobe approach combining fluorescent analogs of both Chol (cholestatrienol; CTL) and SM (pyrene-SM) with a series of StnII Trp mutants was also used to study StnII/bilayer interactions [91]. Comparison of two lipids showing high affinity for SM, and containing an equivalent 1-hydroxyl group, such as oleoyl-ceramide (OCer) or Chol, was the approach chosen [91]. StnII bilayer permeabilization in the presence of OCer or Chol revealed that only the sterol was able to promote the activity of the protein. Experiments using CTL revealed that, in fact, CTL is close enough to StnII to display FRET to the Trp residues of StnII, mostly to Trp110 and Trp114. These experiments also revealed that StnII was able to attract CTL to its vicinity, a behavior extensible to other sterols such as Chol. We speculated that headgroup orientation in SM clusters was different in the presence of Chol and OCer, and that the Chol-induced orientation was preferred by StnII, as it seems to be the case with OlyA [90]. As StnII activation in bilayers is markedly affected by hydrogen bonding [184], it is also possible that Chol and OCer rearrange SM hydrogen bonding differently, which in turn affected StnII-induced bilayer permeabilization. Such rearrangements of SM hydrogen bonding might also affect SM headgroup orientation. These findings provided new details on the process of StnII pore formation as influenced by SM and Chol. The conclusions were similar to those reached for OlyA [90] and further support the interest in studying the mentioned StnIID76S mutant.

## 11. Conclusions

Actinoporins are a fascinating field of study for several reasons. On one hand, they are one of the main components of sea anemones venom. Venoms are a source of valuable information from a biochemical point of view, both for the possible fabrication of antidotes (for the most dangerous species) and the potential use of their components to our benefit [185], such as designing immunotoxins [186,187,188,189,190], sequencing DNA [191], facilitating drugs cell penetration [192] or constructing specific SM sensors [193,194,195]. Furthermore, actinoporins are unique since they can remain stably folded in solution until they encounter a membrane with the adequate composition. This process makes actinoporins an excellent model to study the biophysics of the transformation of a soluble protein to a transmembrane oligomeric structure. They are indeed an excellent example of adaptation to attack a range of prey by binding to an unspecific molecular target as SM. However, the exhaustive research pursued around these proteins revealed a mechanism derived from the multigene family of these toxins, like synergy or the slight differences between isotoxins leading to distinct lytic behavior. Further analysis employing a more complex lipid system must be done to understand the protein–lipid but also the protein–protein interaction network leading to the final pore formation. A better understanding of this metamorphic phenomenon is necessary to reveal the activity details that might be important for future biotechnological applications as biotherapeutics or research tools. In the past decades, researchers have unraveled many of the questions regarding this topic, but some aspects still need to be addressed.

## Abbreviations

BAK	Bcl-2 homologous antagonist/killer
BAX	Bcl-2 associated X protein
CfTX	*Chinorex fleckeri* toxin
Chol	cholesterol
CTL	cholestatrienol
DOPC	dioleyl-phosphatidylcholine
Eqt	equinatoxin
Fra	fragaceatoxin
FRET	fluorescence resonance energy transfer
MACPF	membrane attack complex/perforin family
PFP	pore-forming protein
PLA2	phospholipase A2
POC	phosphorylcholine
POPE-NBD	1,2-dioleoyl-sn-glycero-3-phosphoethanolamine-N-(7-nitro-2-1,3-benzoxadiazol-4-yl)
R6G	Rhodamine 6G
RHA	relative hemolytic activity
RMB	relative membrane binding
SM	sphingomyelin
Stn	sticholysin
wt	wild-type
